# Biomechanics in the tumor microenvironment: from biological functions to potential clinical applications

**DOI:** 10.1186/s40164-024-00591-7

**Published:** 2025-01-11

**Authors:** Hao Peng, Zheng Chao, Zefeng Wang, Xiaodong Hao, Zirui Xi, Sheng Ma, Xiangdong Guo, Junbiao Zhang, Qiang Zhou, Guanyu Qu, Yuan Gao, Jing Luo, Zhihua Wang, Jing Wang, Le Li

**Affiliations:** 1 Department of Urology, Tongji Hospital, Tongji Medical College, Huazhong University of Science and Technology, Wuhan, 430300 China; 2 The Second Clinical School, Tongji Hospital, Tongji Medical College, Huazhong University of Science and Technology, Wuhan, 430300 China; 3 Department of Urology, Renmin Hospital of Wuhan University, Wuhan, 430060 China; 4 Department of Urology, Qinghai University Affiliated Hospital, Qinghai University Medical College, Xining, 810001 Qinghai China; 5 Institute of Reproductive Health, Center for Reproductive Medicine, Tongji Medical College, Huazhong University of Science and Technology, Wuhan, 430030 China; 6 Taikang Tongji (Wuhan) Hospital, 420060 Wuhan, China

**Keywords:** Tumor microenvironment, Biomechanics, Biomechanical target, Extracellular matrix, Stiffness

## Abstract

Immune checkpoint therapies have spearheaded drug innovation over the last decade, propelling cancer treatments toward a new era of precision therapies. Nonetheless, the challenges of low response rates and prevalent drug resistance underscore the imperative for a deeper understanding of the tumor microenvironment (TME) and the pursuit of novel targets. Recent findings have revealed the profound impacts of biomechanical forces within the tumor microenvironment on immune surveillance and tumor progression in both murine models and clinical settings. Furthermore, the pharmacological or genetic manipulation of mechanical checkpoints, such as PIEZO1, DDR1, YAP/TAZ, and TRPV4, has shown remarkable potential in immune activation and eradication of tumors. In this review, we delved into the underlying biomechanical mechanisms and the resulting intricate biological meaning in the TME, focusing mainly on the extracellular matrix, the stiffness of cancer cells, and immune synapses. We also summarized the methodologies employed for biomechanical research and the potential clinical translation derived from current evidence. This comprehensive review of biomechanics will enhance the understanding of the functional role of biomechanical forces and provide basic knowledge for the discovery of novel therapeutic targets.

## Background

The tumor microenvironment (TME) is a highly structured ecosystem that encompasses diverse components, including tumor cells, heterogeneous immune cells, cancer-associated fibroblasts (CAFs), endothelial cells, and the extracellular matrix (ECM), all of which are ubiquitously present across various cancers [1–3]. These constituent elements of the TME do not exist as isolated entities that function independently; rather, a multitude of biochemical signals exist inside and outside the cells within the TME, sustaining cell life activities and mediating intra-/inter-cellular communication. By specifically targeting the inhibition or activation of tumor-associated signaling pathways or genes, it is possible to effectively suppress tumor growth and thereby prolong patient survival [4]. Indeed, targeted therapy has achieved significant progress in the realm of pharmacological treatment, exemplified by tyrosine kinase inhibitors, mTOR inhibitors, VEGF inhibitors, and immune checkpoint inhibitors targeting PD-1/PD-L1 and CTLA-4 [5–10]. Since the early twenty-first century, over 200 targeted therapies have been approved by the FDA, profoundly transforming the landscape of cancer treatment and ushering in the era of precision oncology [11]. Although existing targeted and immunotherapeutic strategies hold significant theoretical potential, they often fail to achieve the anticipated improvements in survival rates in clinical practice owing to low response rates (RR) and issues related to primary or acquired drug resistance [6, 12, 13]. Previous studies have attributed these failures to various mechanisms present within the TME, such as T-cell exhaustion, hypoxia, fibrosis, metabolic dysregulation, and the negative regulatory effects of immunosuppressive cells on immune responses [14–18]. This has led to the identification of several relevant therapeutic targets, which were subsequently explored. However, many of these clinical trials have failed to prolong survival, indicating that our current understanding of the TME may still be limited and superficial [19, 20]. Therefore, expanding our understanding of the tumor microenvironment and identifying new therapeutic targets from diverse perspectives is imperative [21, 22].

**Fig. 1:**
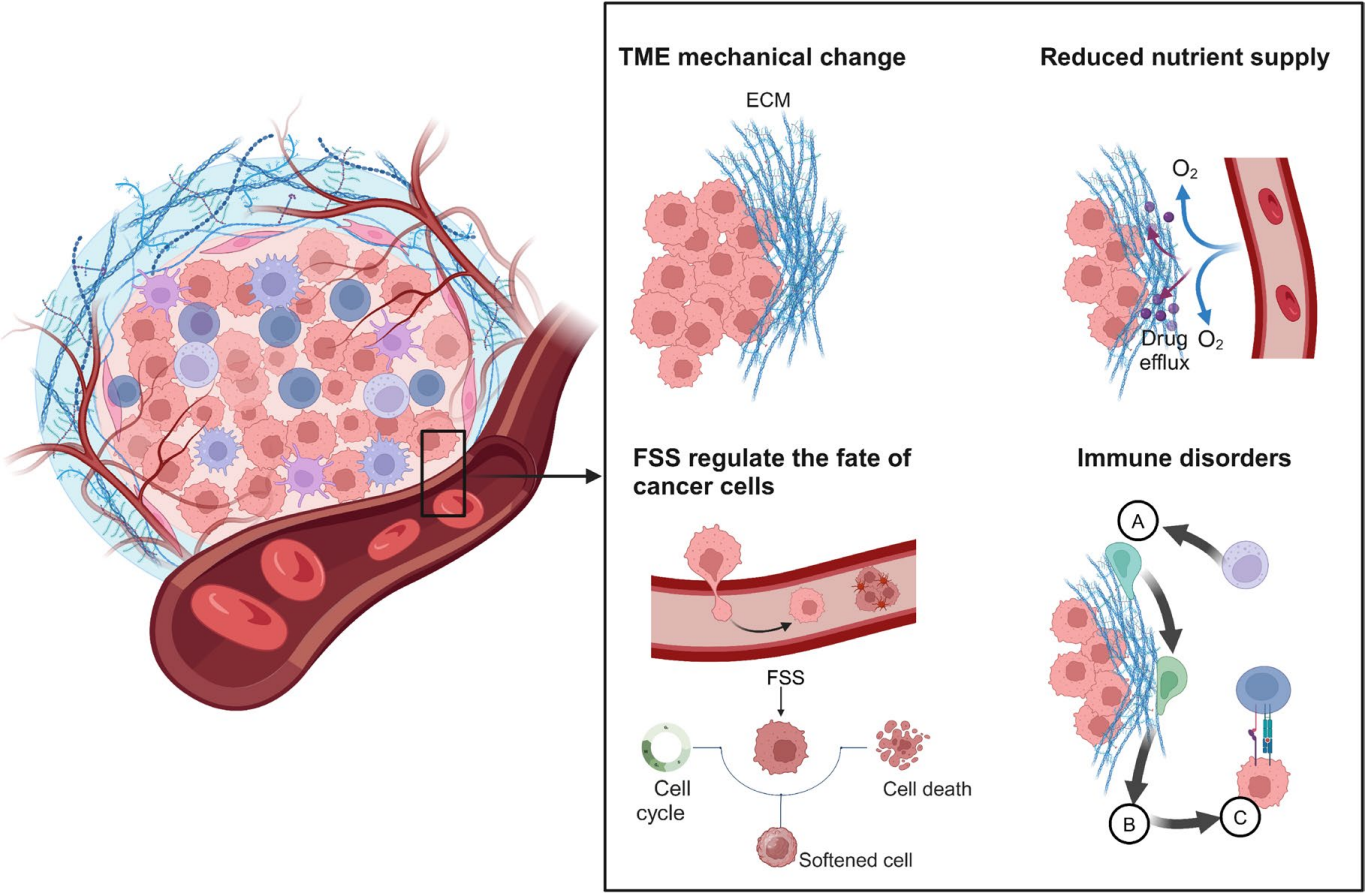
The complex mechanical perturbations within the tumor microenvironment. The TME is an intricate ecosystem comprising tumor cells, endothelial cells, stromal cells, diverse immune cells, and the extracellular matrix. In this context, the stiffness of cancer cells decreases, while the stiffness of the ECM increases, leading to an elevation in IFP. This, in turn, impedes the delivery of nutrients and oxygen, and promotes the accumulation of metabolic waste, ultimately obstructing the delivery of anti-cancer drugs to the tumor region. The cancer cells of the primary tumor site invade and enter the circulatory system to form CTCs. On the one hand, other cells in the bloodstream protect CTCs from FSS by encapsulating them; on the other hand, tumor cells also adjust their own stiffness to adapt to FSS. In addition, FSS also regulates the cell cycle of CTCs. Furthermore, the mechanical disturbances within the TME also compromise the infiltration of immune cells and their cytotoxic capabilities through inducing immune cell exhausted and unstable immune synapses. *TME* tumor microenvironment, *ECM* extracellular matrix, *IFP* interstitial fluid pressure, *CTCs* circulating cancer cells, *FSS* fluid shear forces

Recent evidence indicates that within the various cells that comprise the TME, as well as blood vessels, lymphatic vessels, and the ECM, not only the transmission of biochemical signals but also the influence of mechanical signals generated by the physical properties of these components and their interactions exist (Fig. 1) [23]. The activation or dysregulation of these biomechanical signals may play crucial roles in distant tumor metastasis, immune evasion, and resistance to targeted therapies [24]. For example, a high-stiffness ECM exerts mechanical stimuli on tumor-infiltrating CD8^+^ T cells. This mechanical signal is sensed by the mechanoreceptor Piezo1 on the surface of T cells, which subsequently mediates Ca^2+^ influx and the activation of downstream T-cell receptor (TCR) pathways [25]. Through the amplification of the Piezo1/CaMKII/CREB/Osr2 signaling cascade, this process ultimately promotes T-cell exhaustion and resistance to immunotherapy [25]. As a result, the inhibition of OSR2 signaling significantly potentiated cancer immunotherapy [25]. These findings provide new insights for the treatment of certain hard-textured solid tumors, including hepatocellular carcinoma (HCC), prostate cancer, and triple-negative breast cancer.

Cells are not merely passive recipients of mechanical forces; rather, they can actively alter their physical properties to adapt to the varying stiffness of the ECM or transmit different mechanical signals to other cells. Cancer cells, as the most heterogeneous cells within the TME, can actively adopt a softer phenotype during metastasis to reduce physical damage from shear forces exerted by blood flow [26, 27]. Upon colonization, they can extend pseudopodia and increase their physical stiffness to enhance focal adhesion (FA) with the ECM. Consequently, integrins, which are key molecules determining the cytoskeletal architecture of cancer cells, are promising targets for the treatment of cancer progression [28].

Given the significant role of biomechanics in cancer research and the relative unfamiliarity with this emerging field, this review aims to revisit important advancements over the past few decades. We first introduced the variations in tumor cell stiffness and their corresponding effects on cellular fate. We subsequently provide a detailed discussion of the interactions among tumor cells, immune cells, and the ECM, summarizing promising therapeutic targets identified in these interactions. We also compiled common measurement techniques and methodologies for researchers in the field of biomechanics. Finally, we present an overview of current preclinical and clinical trials targeting biomechanical pathways for clinicians and drug developers.

## Variations in the mechanical properties of tumors

### Alterations in the stiffness of cancer cells

The diversity of tumor cell stiffness is a significant manifestation of cancer heterogeneity. Stiffness refers to the ability of an object to resist local pressure-induced deformation. In materials science, stiffness is determined by the elastic modulus and topological structure, with the elastic modulus typically quantified by Young’s modulus [29]. This quantifies the ratio of the force applied to a cell or material (under compression and tension) to the resulting strain [29]. Given a constant topological structure, a higher elastic modulus corresponds to increased stiffness. Therefore, in the context of biophysics, the cell elastic modulus typically refers to stiffness [30]. Quan et al. compared the stiffness of eight types of solid cancer cells with that of normal cells and reported that most stiffness variations ranged from 20 to 80% [31]. For example, the stiffness of breast cancer cells ranges from 0.1 to 0.4 kPa, whereas normal breast epithelial cells present greater stiffness values (0.2–0.9 kPa). The stiffness of various bladder cancer cells ranges between 0.4 and 5.2 kPa, whereas normal bladder cells have significantly greater stiffness (10–16 kPa) [31]. Consistent with these findings, Lin et al. reported that the stiffness of Ha-RasV12-transformed cells was lower than that of normal cells [32]. Kwon et al. measured the stiffness of breast cancer, cervical cancer, and lung cancer cells, as well as their normal counterparts, via atomic force microscopy (AFM) under identical conditions [33]. They reported that the stiffness of normal cervical cells (Ect 1/E6E7) was 48.8 ± 3.3 kPa, whereas the stiffness of cervical cancer cells (HeLa, SiHa, and Caski) ranged from 21.1 to 26.7 kPa, exhibiting the greatest reduction in stiffness among the three types of cancer [33]. These studies consistently suggest that the reduction in cell stiffness during the process of carcinogenesis may be a common phenomenon and that the decrease in magnitude of cancer cells is linked to their tissue origins, which could inspire new biomechanical-based approaches for the diagnosis of cancer. In addition to the high heterogeneity in stiffness among cancer cells from different tissues, variations in stiffness among the same type of cancer cells also reflect different stages of cellular fate. Mandal et al. confirmed that a decrease in the stiffness of bladder urothelial cells is associated with an increase in cancer grade [34]. This may be due to a greater proportion of cancer stem cells (CSCs) at higher cancer grades, which exhibit lower stiffness than typical tumor cells do. Babahosseini et al. reported that the elastic modulus of CSCs derived from a spontaneously transformed mouse ovarian cancer model was reduced by 46, 61, and 72% compared with that of late-stage tumor cells, moderate-grade tumor cells, and normal cells, respectively [35]. Similarly, in breast cancer, colorectal cancer, and liver cancer, CSCs also display 20–75% lower stiffness than nonstem cancer cells do [36–38]. However, it should be acknowledged that the stiffness of cells measured under different methods and conditions can vary hundreds of times [39]. The differences in measurement techniques and conditions complicate the comprehensive analysis of the stiffness of cancerous versus normal cells. Therefore, it is essential to establish standardized measurement protocols and develop a comprehensive database of stiffness for tumor cell lineages to enhance our understanding of the relationship between cancer cell stiffness and their cellular states.

One of the purposes of reducing the stiffness of cancer cells is to achieve enhanced invasive capabilities. A study involving various ovarian cancer cell lines revealed that the migration and invasion abilities of cancer cells were inversely proportional to cell stiffness; by pharmacologic inhibition of myosin II, both cell stiffness and invasive potential could be decreased [40]. Increasing the expression of the metastasis suppressor TβRIII/β-glycan can, in turn, similarly increase cell stiffness [40]. Similar results have also been reported in melanoma, prostate cancer (PCa), and tongue squamous cell carcinoma [41–44]. Palmieri et al. utilized AFM to measure the stiffness of SW 480, a colorectal cancer cell line composed of two cell populations: elongated cells and fewer rounded cells [45]. They reported that the stiffness of highly metastatic and elongated cells (500 Pa) was lower than that of less metastatic and rounded cells (1000 Pa) [45]. Given the extensive and complex mechanical deformations involved in the metastasis of tumor cells, it is generally accepted that cells with a lower stiffness exhibit better deformability, whereas those with a higher stiffness can generate greater local adhesive forces to transmit mechanical stimuli. This explains why tumor cells with lower stiffness tend to have greater metastatic potential. Furthermore, the stiffness of tumor cells influences the sites of colonization after metastasis. Tang et al. reported that breast cancer cells with stiffness greater than that of their parental cells exhibited a stronger tendency for bone metastasis, whereas breast cancer cells with lower stiffness demonstrated a greater tendency for brain metastasis, aligning with the lower stiffness of brain tissue [46].

Notably, some controversy remains regarding the relationship between the stiffness of tumor cells and their invasive capabilities. For example, more invasive PCa cell lines (22 RV1, LNCaP, DU 145, and PC 3) do not exhibit lower contractility, cell stiffness, or motility than less metastatic PCa cells do [47, 48]. This finding is consistent with Darling et al.’s observation that there is no significant correlation between stiffness and invasiveness in osteosarcoma tumor cells [49]. Consequently, some researchers argue that tumor metastatic potential cannot be simply measured by cell stiffness alone. The relationship between tumor cell stiffness and invasiveness is complex and may involve other mechanical properties that collectively determine the metastatic capacity of tumor cells. Kashani et al. proposed a novel concept known as the migration index [50]. This index calculates the strain energy stored in cells of varying volume and stiffness on the basis of contractility via a mathematical model to ascertain migratory ability. According to this model, there exists a critical stiffness-to-volume ratio for cells that maximizes the migration index [50].

Interestingly, the ability of metastatic tumor cells to reduce their stiffness is likely due to a combination of both passive and active mechanisms. During cancer metastasis, tumor cells are subjected to fluid shear forces (FSS) exerted by the interstitial fluid, ascites, lymphatic vessels, and blood vessels [51]. Notably, when tumor cells traverse the basement membrane barrier, diffuse into blood vessels, and become circulating tumor cells (CTCs), they may experience an FSS as high as 3000 dyn/cm^2^ [52]. FSS can damage the cell membranes of tumor cells that are unable to adapt, inducing membrane perforation. If these perforations cannot be rapidly repaired, the cytoplasmic contents, including ions, ATP, and even organelles, leak out through the membrane, leading to cell death [53]. Moreover, tumor cells under high FSS produce more reactive oxygen species (ROS), which increases oxidative stress and contributes to cell mortality [54]. To better survive under such stress, tumor cells primarily employ two strategies. On the one hand, they can adhere to neutrophils, monocytes, and platelets, with platelets serving as their most important "shield" to reduce the direct impact of FSS [55–57]; on the other hand, they "soften" themselves to better buffer the effects of FSS. This "softening" primarily manifests as an increase in surface area, thickening of the cortex, and softening of the nucleus [27]. Favero et al. reported that T24 bladder cancer cells exhibited a significant increase in surface area when induced by FSS at a flow rate of 0.03 ml/min under in vitro culture conditions [58]. Xu et al. reported that FSS can promote histone acetylation in CTCs, potentially increasing the nuclear size and contributing to nuclear softening [26]. These findings indicate that the biomechanical properties of cancer cells are not static; rather, they undergo dynamic changes in response to varying external environments. Simultaneously, changes in the mechanical properties of cancer cells are accompanied by alterations in their proliferative potential and cell cycle. High shear stress can directly decrease the percentage of tumor cells in the G0/G1 phase while increasing the proportion of cells in the S/G2 phase by regulating the expression of proteins related to the cell cycle and lipid metabolism, thereby inhibiting the proliferative potential of cancer cells [59]. This observation provides a reasonable explanation for how cellular biomechanics specifically regulate tumor cell fate.

### Alterations in the stiffness of the ECM

The increased stiffness of the ECM is also a significant characteristic of the TME. The ECM is a noncellular component present in all tumor tissues and is primarily composed of water, proteins, and polysaccharides. The ECM can be divided into the basement membrane and the stromal matrix. The basement membrane is primarily composed of laminin and collagen, which serve to separate epithelial and endothelial cells from the stromal matrix [60]. The stromal matrix is mainly composed of various proteins, including collagen, elastin, proteoglycans, hyaluronic acid (HA), fibronectin, osteopontin, and fibrillin, which collectively maintain the mechanical integrity of tissues [61]. Proteoglycans provide compressive strength through their glycosaminoglycan chains that bind water, whereas collagen types I, III, and V, along with fibronectin, serve as the primary proteins for forming fibrous structures [60]. These proteins facilitate cell adhesion to the ECM and participate in intercellular signaling through the formation of FA [62]. Therefore, the stiffness of the ECM is primarily determined by the concentration, assembly, and crosslinking density of these proteins. During cancer progression, the ECM undergoes profound remodeling mechanisms, including changes in chemical composition, biological modifications, and alterations in physical properties, which jointly contribute to the increased stiffness of the ECM in the TME [63]. Numerous studies have demonstrated that ECM stiffness increases in a wide range of tumors, including breast, lung, pancreatic, and brain tumors [64–67]. In general, the stiffness of the ECM in cancerous tissues is typically 8 to 10 times greater than that in normal tissues from the same organ [60, 68]. The increased quantity and cross-linking of proteins such as collagen I/III and glycoproteins in the ECM are significant contributors to increased stiffness [69–71]. Collagen cross-linking is a multi-step process. Various cells within the TME, including fibroblasts, can secrete members of the lactate oxidase (LOX) family, which catalyze the oxidative deamination of lysine and hydroxylysine residues, facilitating the formation of aldehydes that can spontaneously cross-link collagen [72]. Paszek et al. reported that increased expression of LOX leads to excessive collagen cross-linking, resulting in breast cancer ECM being 5 to 25 times stiffer than surrounding normal breast tissue [73]. Additionally, CAFs can secrete matrix metalloproteinases (MMPs) to remodel the ECM, creating new spaces for tumor cell proliferation and metastasis [74]. More importantly, the cleavage of the ECM by MMPs can also generate molecules that promote tumor progression and metastasis [75]. These products can enter the bloodstream as biomarkers for cancer, potentially serving as new tools for the clinical diagnosis of high-risk metastatic patients [75]. Increased advanced glycation end products (AGEs) can also increase the stiffness of the ECM in cancer, further promoting tumor development [76]. Fan et al. reported that type 2 diabetes leads to the accumulation of AGEs in the ECM of hepatocytes, thereby promoting the occurrence, proliferation, and invasion of HCC through the integrin-β1–tensin-1–YAP mechanotransductive pathway [77]. In addition to changes in the composition of the ECM, mechanical stress caused by tumor proliferation and cell contraction can also stretch or compress the ECM, leading to alterations in ECM stiffness [73].

ECMs with varying stiffnesses exert significantly different stimuli on tumor cells, thereby regulating their proliferation, autophagy, and metastasis. Wu et al. discovered that a stiff ECM can activate the Akt signaling pathway in tumor cells, promoting the loading of GTP onto Rab8 and driving the secretion of extracellular vesicles, which in turn activates the Notch pathway in adjacent cancer cells, facilitating tumor growth [78]. Zhang et al. found that a stiff ECM can activate the integrin-GSK3β-FTO-mTOR axis, enhancing tumor cell anabolic metabolism through mTORC1 and thereby providing nutrients for rapid tumor growth [79]. Conversely, a lack of sufficient stimuli from ECM stiffness can induce autophagy and metabolic reprogramming in tumor cells, which in turn conveys the resilience of tumor cells to anoikis [79]. The stiffness of the ECM is also crucial for the formation of CSCs. When the stiffness of the CSC niche increases, cytoplasmic TAZ in tumor cells is activated by mechanical forces from the microenvironment and translocates into the nucleus, where it undergoes phase separation with the stem cell transcription factor NANOG, promoting the expression of associated stem cell genes and maintaining CSC-related properties [80]. Using this principle, culturing tumor cells in fibrin gels with a stiffness of approximately 100 Pa can increase the efficiency of selecting CSCs [81]. Other ECM components that have similar effects include type I collagen and HA, which can induce the transition to a CSC phenotype by activating integrins or the transforming growth factor-β (TGF-β)-Snail signaling pathway [82–85].

A stiff ECM can also promote tumor invasion and metastasis through mechanisms that induce epithelial‒mesenchymal transition (EMT), independent of the promotion of CSC phenotype transformation. EMT is the process by which epithelial cells acquire mesenchymal characteristics and enhanced motility, along with cellular and nuclear deformations, and is often considered a prerequisite for the invasion and metastasis of cancer cells [86]. When tumor cells are subjected to mechanical stimuli from a stiff matrix, the mechanomediator TWIST1 is released from its cytoplasmic binding partner G3BP2 and translocated to the nucleus, where it promotes EMT in response to increasing matrix stiffness [87]. EMT can downregulate the enrichment of ezrin in lamellipodia and reduce the formation of lamellipodia in tumor cells, thereby transforming tumor epithelial cells into a mesenchymal migratory phenotype and facilitating tumor metastasis [88]. Within the stiffness range of 10 kPa, breast cancer cells exhibit enhanced adhesion and invasion capabilities as ECM stiffness increases, accompanied by more elongated and polarized cell morphologies. However, this enhancement peaks at an ECM stiffness of 10 kPa and no longer increases beyond that point [50, 89]. Interestingly, the maximum stiffness threshold at which the ECM promotes tumor cell metastasis varies among different types of cancer cells: for PC3 cells, this threshold is 46.7 kPa, whereas for LNCaP cells, it is 0.7 kPa [90]. The source of this disparity remains unclear; it may be related to the different metastatic patterns of tumor cells. Common metastatic patterns involve either the migration of individual tumor cells or collective migration in clusters. A relatively high stiffness ECM (>10 kPa) stimulates PC3 cells to promote single-cell metastasis through YAP/TAZ nuclear translocation, whereas a relatively low stiffness ECM (0.5 kPa) induces metastasis via cellular clusters [90].

## Biomechanics of antitumor immunity in the TME

### Tumor cells, immune cells and immune synapses

**Fig. 2:**
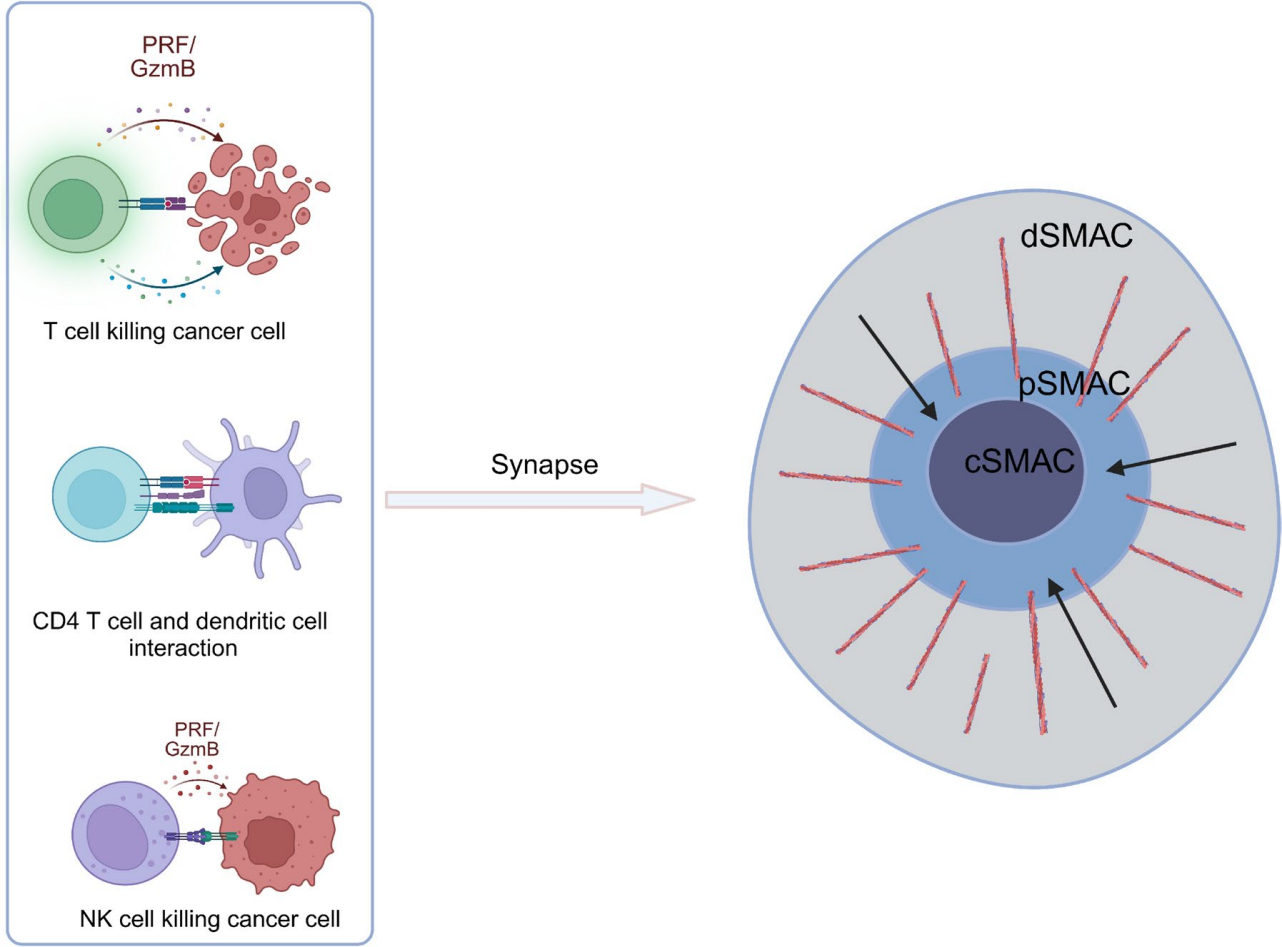
Schematic diagram of the immune synapses. The immune synapse is the structural basis for lymphocytes to receive antigen presentation and effector functions by secreting lytic granules. The *arrow* on the *right panel* indicates the direction of retrograde F-actin flow. *dSMAC* distal supramolecular activation cluster, *cSMAC* central supramolecular activation cluster, *pSMAC* peripheral supramolecular activation cluster

Tumor immune responses depend on the formation of immune synapses (IS), and mechanical signaling between tumor cells and immune cells also occurs through this structure. IS is defined as the intercellular contact between at least one immune cell and another cell and is characterized by protein aggregation in micrometer-scale regions (Fig. 2) [91]. Traditionally, the IS in the TME consists of two main types: the "stimulatory" IS formed between immune cells and antigen-presenting cells (APCs) and the "cytotoxic" IS established between CD8^+^ T/NK cells and target cells [92]. This review focuses primarily on the "cytotoxic" IS formed between CD8^+^ T/NK cells and target cells.

The process of IS formation between T cells and target cells includes adhesion, lytic granule polarization, and target disengagement phases [93]. After T cells recognize antigens, anchor adhesion molecules, undergo costimulatory activation and a certain degree of mechanical stimulation, intracellular lytic granules move along microtubules toward the microtubule organizing center (MTOC) and are transported to the central supramolecular activation cluster (cSMAC), which contains antigen receptors and associated signaling proteins [94]. Upon binding to target cells, T cells form a cytotoxic synapse, polarizing lytic granules to the synaptic cleft, where they fuse with the cell membrane of the target cell of appropriate stiffness, resulting in a perforation effect [95]. The formation of cytotoxic IS in NK cells is similar, but granule release depends on the relative strength of the activating and inhibitory signals within the IS. When activating signals dominate, NK cells undergo MTOC polarization, leading to subsequent degranulation. Conversely, when inhibitory signals prevail, MTOC polarization is hindered, preventing degranulation [96]. However, tumor cells can evade direct killing and achieve immune escape by remodeling their cytoskeleton and reducing their cell stiffness through decreased contractility of actin II, thereby inhibiting the normal formation of CD8^+^ T/NK IS [97–99]. Additionally, tumor cells can adjust the cytotoxic effects of CD8^+^ T/NK cells by altering the cytoskeletal and membrane dynamics within tumor cells. For example, treating melanoma cells with cytochalasin D to reduce Filamentous actin (F-actin) significantly decreases the release of granzyme B from cocultured CD8^+^ T cells [99]. Similarly, gene knockout of ACAT1, which reduces cholesterol levels in tumor cell membranes, can significantly decrease cancer cell stiffness, thereby inhibiting T-cell-mediated cytotoxicity [100]. Mechanical stimulation of tumor cells by CD8^+^ T/NK cells activates the mechanoreceptor PIEZO1 on their surface, mediating the remodeling of their intracellular cytoskeleton and thus modulating their cytotoxic functions [101]. Some metabolites present in the tumor microenvironment, such as lysophosphatidic acid, can also disrupt IS formation by impairing F-actin accumulation in CD8^+^ T cells and altering microtubule dynamics, allowing tumor cells to evade death [102]. In summary, the IS plays a crucial role in tumor immunology. However, our understanding of the relationship between the IS and biomechanics, particularly how cytoskeletal components such as actin and microtubules contribute to IS formation, remains limited and requires further investigation. Additionally, the effects of other membrane factors, such as various membrane proteins and lipids, on IS formation are not fully understood, highlighting the need for more research to address key issues regarding IS and their interactions with anticancer immunity.

### ECM stiffness and immune cells

The ECM significantly influences immune cell function. Historically, this influence was thought to stem primarily from biochemical factors such as metabolism and hypoxia within the ECM [103]. However, recent studies have shown that the physical properties of the ECM are also crucial to the functionality of immune cells. As early as 2012, researchers discovered that hydrogels with higher stiffness stimulated greater proliferation and activation of T cells, including CD4^+^ and CD8^+^ T cells [104–107]. The use of these more stimulated and activated CD8^+^ T cells for adoptive transfer immunotherapy significantly extends the survival of tumor-bearing mice [107]. Nevertheless, there are discrepancies between in vitro experiments using ECM-mimicking hydrogels and in vivo experiments. Zhang et al. found that increased stiffness of the ECM in advanced HCC activated the mechanoreceptor Piezo1 on T cells, promoting Ca^2+^ influx and enhancing CD8^+^ T-cell exhaustion through the CaMKII/CREB/Osr2 axis, ultimately limiting the efficacy of immunotherapy [25]. These seemingly contradictory findings may provide a plausible explanation: the impact of mechanical stimulation on T-cell function may resemble that of antigen stimulation, where a certain degree of short-term mechanical stimulation is necessary and beneficial for T-cell activation. However, prolonged high-intensity mechanical stimulation can lead T cells to enter an exhausted state prematurely, which is detrimental to antitumor immunity [108]. In addition to direct mechanical signaling, a stiff ECM can obstruct the delivery of oxygen and nutrients by compressing blood vessels and lymphatics within tumors while impeding waste clearance, leading to a metabolic environment characterized by hypoxia and lactic acid accumulation, which mediate T-cell dysfunction [63]. As previously mentioned, the increased stiffness of the ECM primarily arises from densely interconnected collagen. These tightly arranged barriers significantly inhibit the infiltration of CD8^+^ T cells, resulting in a "desert-like" infiltrative phenotype in the TME, which further promotes the failure of targeted immunotherapy against CD8^+^ T cells [109–111].

Similar to that of T cells, the antitumor function of NK cells also requires a certain degree of stiffness stimulation from the ECM. Within the range of 2–50 kPa, increasing ECM stiffness enhances NK cell activity and promotes the formation of stable IS with target cells, thereby increasing NK cell degranulation activity [97]. In contrast, soft ECM inhibits the polarization of the MTOC, granule polarization, and F-actin accumulation, leading to the formation of unstable IS between NK cells and tumor target cells, resulting in reduced degranulation [97]. Notably, the increased immune activity of NK cells in response to mechanical stimulation from the ECM results in a "bell-shaped" response, and only a subset of the NK cell population can respond to changes in stiffness [112].

The stiffness of the ECM also affects the migration patterns and inflammatory (M1 or M2) phenotypes of macrophages. Sridharan and colleagues reported that high-stiffness polyacrylamide gels (323 kPa) promote a pro-inflammatory macrophage phenotype, whereas soft (11 kPa) and moderately stiff (88 kPa) polyacrylamide gels induce an anti-inflammatory phenotype [113]. This may be due to high-stiffness polyacrylamide gels activating Piezo1-mediated Ca^2^⁺ influx, which subsequently activates the downstream NF-κB and STAT6 signaling pathways, mediating the formation of the pro-inflammatory phenotype in macrophages [114]. Interestingly, 3D collagen-rich environments promote M2 polarization of macrophages and enhance their immunosuppressive phenotype, thereby reducing the efficiency of attracting cytotoxic T cells and increasing the ability to inhibit T-cell proliferation [115]. Under a stiff ECM, tumor-associated macrophages can also consume arginine, synthesize proline, and secrete ornithine, which suppresses the function of CD8⁺ T cells [116]. These macrophages can further promote tumor metastasis by stimulating stromal cells to overexpress lysyl hydroxylase and LOX, mediating collagen cross-linking and increasing the stiffness of the ECM [117]. These seemingly contradictory results may arise from differences in matrix materials and culture models (3D vs. 2D), suggesting that simple in vitro hydrogel models may not be ideal for studying the biomechanics of the TME.

In summary, the impact of ECM stiffness on tumor immune responses is highly complex and may depend on the cumulative effects of its promotion or inhibition of different immune cell functions. However, most evidence supporting the notion that increased matrix stiffness enhances immune responses comes from in vitro experiments, which contradicts the observation of immune suppression mediated by increased ECM stiffness in the TME. These findings suggest that the rigid materials currently used in vitro are still far from accurately representative of the true TME, highlighting the need for further development of new models to explore the relationship between ECM stiffness and tumor immunity.

## Mechanisms of mechano-transduction in the TME

**Fig. 3:**
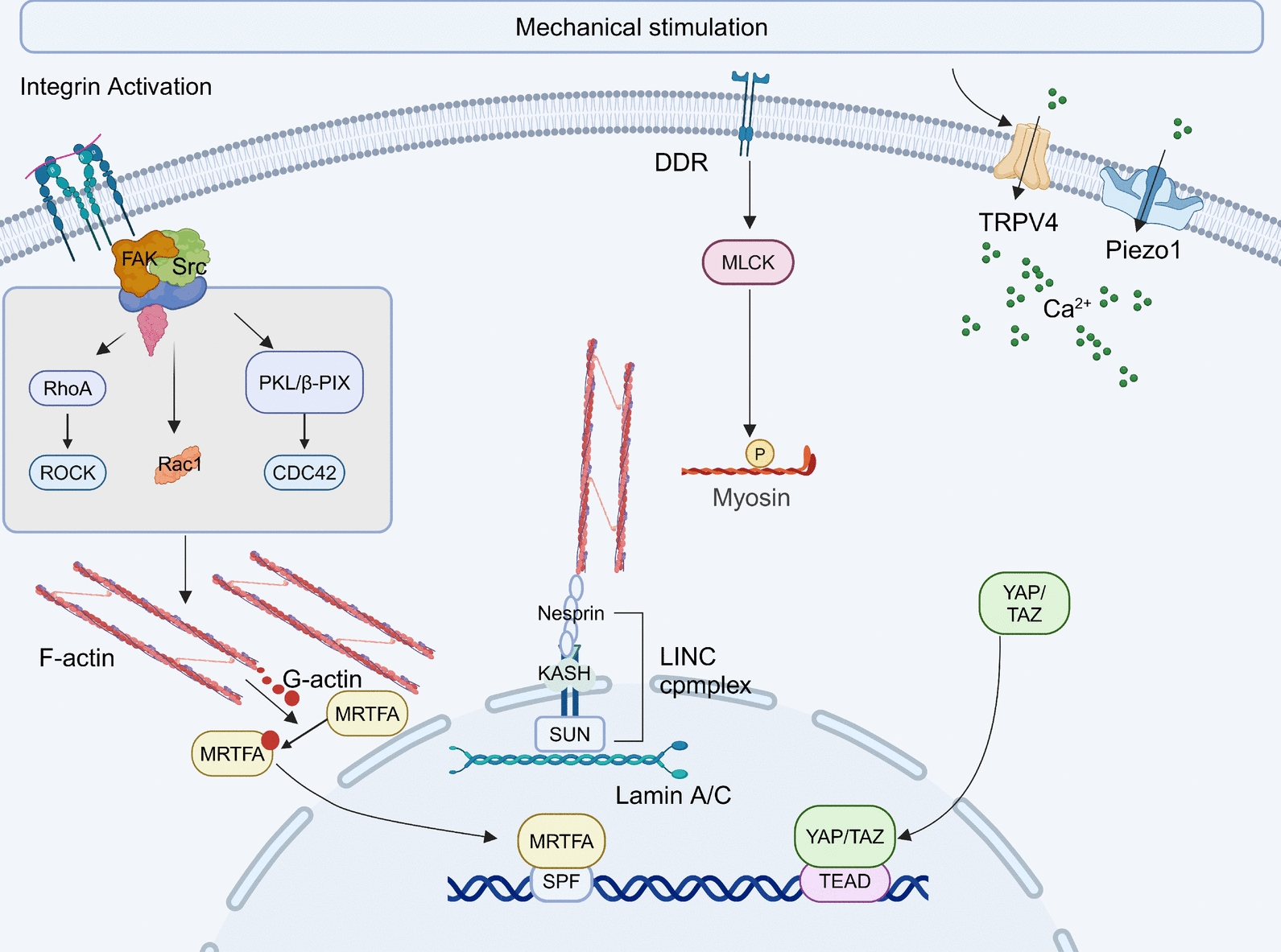
Overview of cellular mechanotransduction pathways. Mechanical force or a stiff ECM stimulates membrane mechanoreceptors, triggering a cascade of downstream reactions: (i) Activated integrins anchor to the ECM, forming focal adhesions and recruiting FAK and SRC to form a complex that activates the Rho protein family, thereby altering the disassembly and assembly of actin filaments. The polymerization of F-actin reduces the availability of G-actin, decreasing its binding to MRTFA. MRTFA then translocates to the nucleus and co-participates with SRF in gene transcription; (ii) Phosphorylated and activated DDR interacts with MLCK, thereby activating myosin; (iii) The cation channels TRPV4 and Piezo1 are activated by mechanical stimuli, promoting Ca2^+^ influx; (iv) The unphosphorylated YAP/TAZ translocates to the nucleus, where it binds to the TEAD family members to form a functional transcriptional complex, promoting the expression of downstream genes; (v) The cytoskeleton can transmit extracellular physical stimuli directly to the nucleus by connecting one end to adhesion structures on the cell membrane and the other end to the nuclear lamin proteins A/C through the LINC complex. *ECM* extracellular matrix, *LINC* the linker of nucleoskeleton and cytoskeleton complex

Extracellular mechanical stimuli are first transmitted to mechanoreceptors on the cell surface, including integrins, discoidin domain receptors (DDR), and ion channels (including the PIEZO family of mechanically activated cation channels and TRPV4) (Fig. 3) [118]. Through a series of adaptor proteins and second messengers, these physical cues are subsequently translated into biochemical signals, inducing intracellular events such as Ca^2+^ influx, cytoskeletal rearrangement, and transcriptional regulation [119–122]. These biomechanical signals ultimately exert significant effects on tumor cell invasion and metastasis, as do interactions between tumor cells, immune cells, and the ECM.

### Cellular mechanoreceptors

Integrins are widely distributed on the surfaces of tumor cells, immune cells, and fibroblasts, serving both as molecular switches for mechanosensing and as bidirectional gateways for signal transduction. They represent a crucial interface for the interaction between cells and their surrounding environment [123]. The integrin family comprises numerous molecules, each of which activates diverse signals and performs various functions. These functions primarily include (1) mediating cell adhesion through the recruitment of adaptor proteins such as vinculin, talin, and focal adhesion proteins [119]; and (2) receiving mechanical signals that activate downstream CDC42-Rho signaling pathways, thereby remodeling cytoskeletal dynamics [124, 125]. Consequently, integrin signaling can drive multiple functions of CSCs, including tumor initiation, epithelial plasticity, metastatic reactivation, and resistance to immunotherapeutic targeting [123, 126, 127].

DDRs are located primarily on the surface of tumor cells and represent a unique class of receptor tyrosine kinases (RTKs) that can bind to collagen fibers and sense mechanical forces, mainly DDR1 and DDR2 [128]. Their primary function is to sense the stiffness of the ECM, forming a positive feedback loop that amplifies the relevant mechanotransduction signals, thereby promoting ECM remodeling and enhancing ECM fibrosis, which in turn inhibits T-cell immune infiltration [121].

Piezo1 has emerged as the most important mechanosensitive receptor for T-cell mechanosensing in recent years [129]. When mechanical forces are transmitted to the T-cell membrane, they increase membrane tension, which subsequently leads to significant increases in membrane curvature and cross-sectional area, stimulating further opening of the non-selective cation channels Piezo1/2 [130]. Activation of Piezo1 is considered a necessary prerequisite for T-cell activation; however, sustained excessive activation of Piezo1 can mediate overactivation of T-cell OSR2 transcriptional activity and inhibit the secretion of cytokines such as IFN-γ and Prf1, ultimately leading T cells to adopt an exhausted phenotype [25, 108].

TRPV4 is another important mechanosensitive channel that relies on Ca^2^⁺ influx, and it is widely expressed on the surfaces of tumor cells and immune cells [131–133]. However, its effects on tumor cell proliferation and metastasis, as well as its influence on immune cell functions, have not yet yielded consistent conclusions. Future research is needed to explore the role of TRPV4 in the TME.

### Mechano-transduction signaling and effects

Although the specific downstream signaling pathways activated by different mechanosensitive receptors vary, they primarily include cascades that mediate Ca^2^⁺ influx and/or trigger downstream kinases, such as RhoA GTP-PAK-ROCK (integrins), RTK-MLCK (DDR), and CaMKII-MLCK (Piezo1) [121, 122, 134]. Subsequently, the activated kinases promote or inhibit the activity of transcription factors, ultimately participating in the maintenance of tumor stemness, proliferation, metastasis, and interactions with immune cells within the TME. In this process, cytoskeletal remodeling plays a central role. On the one hand, the cytoskeleton can transmit extracellular physical stimuli directly to the nucleus by connecting one end to adhesion structures on the cell membrane and the other end to the nuclear lamin proteins A/C [135]. Significant remodeling of the cytoskeleton strongly affects the efficiency of mechanical signal transduction and ultimately influences transcriptional stimulation within the nucleus. Among the most important transcription factors directly influenced by the cytoskeleton identified thus far are YAP/TAZ and OSR2 [25, 135]. On the other hand, the cytoskeleton is a crucial component for maintaining the three-dimensional architecture of the cell. Changes in the cytoskeleton due to remodeling can directly lead to alterations in cell morphology and physical properties, thereby affecting cell stemness and metastatic potential [135].

## Methods and techniques for biomechanical research

**Fig. 4:**
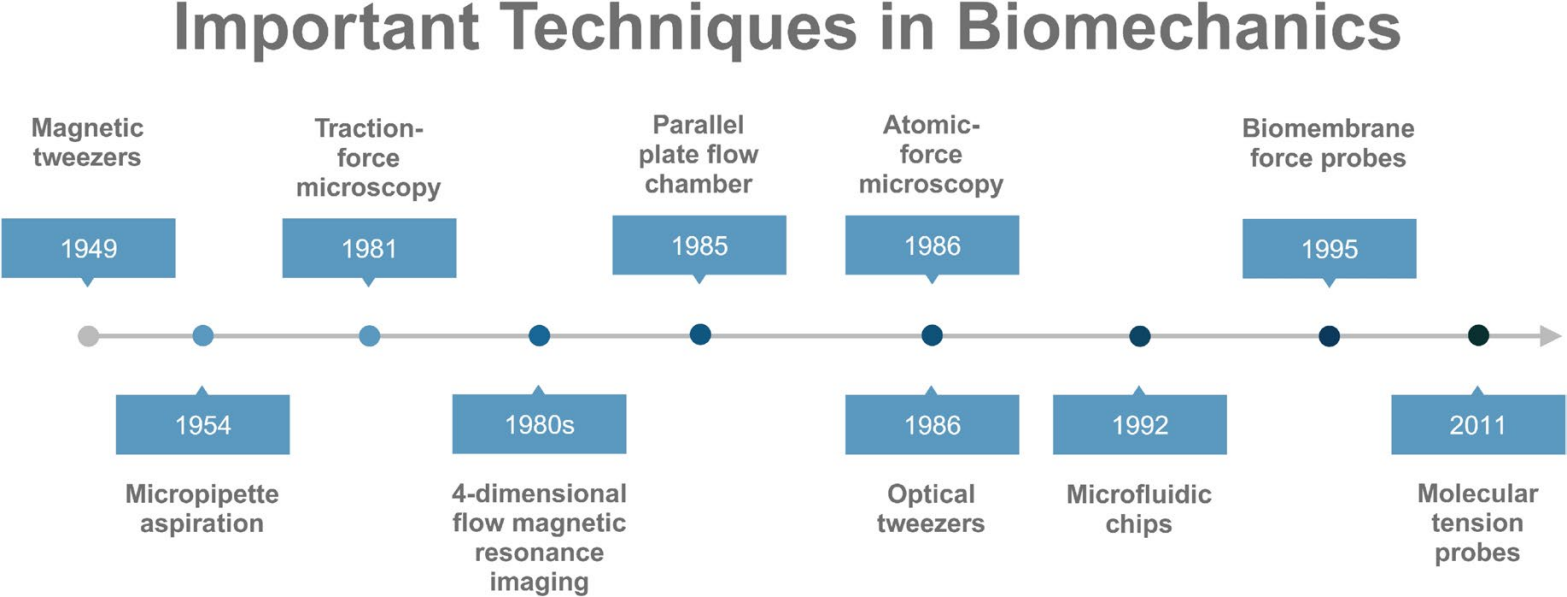
The timeline of important techniques in biomechanical research

Importantly, biomechanics, as an interdisciplinary field, has made significant advancements driven by the development and improvement of interdisciplinary technologies and methodologies from fields such as biophysics and biochemistry [136, 137]. Over the past decade, technologies such as single-molecule force measurements and microimaging have enabled us to overcome the limitations of cellular-level studies, providing deeper insights into how mechanics broadly and profoundly participate in or govern biological activities at the subcellular level (Table 1; Fig. 4) [138–148]. For example, Duan and colleagues developed a double-stranded DNA (dsDNA) probe modified with specific ligands for cellular mechanoreceptors [149]. When cells are seeded onto surfaces coated with these dsDNA probes, surface receptors such as integrins bind to the ligands on the double helix and apply force. Forces exceeding the mechanical tolerance of the double helix lead to its rupture, exposing the activator (the bottom strand), which triggers the endonuclease activity of Cas12a and cleaves the fluorescent single-stranded DNA reporter gene, resulting in the emission of a fluorescent signal. DNA-based probes also include the recently reported ForceChrono, which overcomes the limitations of in vitro single-molecule force spectroscopy by enabling direct measurements of single-molecule force magnitude, duration, and loading rates in dynamic cellular environments [150]. Using this technique, Hu et al. successfully measured integrin force loading rates of 0.5–2 pN/s and durations ranging from tens of seconds in nascent adhesions to approximately 100 s in mature focal adhesions [150]. However, there are currently no DNA-based probes capable of measuring single-molecule forces in cells within living animals or even in humans, which remains a major research direction in the field. In addition, dynamic super-resolution imaging technologies combined with specific fluorescent dyes enable researchers to monitor real-time forces corresponding to various biological activities [151, 152]. In addition, embedded single-molecule force manipulation techniques allow for the active and precise application of different magnitudes of force, facilitating the observation of different biological phenomena induced by varying mechanical stimuli [153]. In the future, artificial intelligence and machine learning are expected to enhance measurements of biomechanics. In fact, there have been attempts to construct models capable of predicting force magnitudes on the basis of machine learning results from images of cytoskeletal proteins, given the close relationship between the cytoskeleton and cellular mechanical properties [154]. However, limitations in the understanding of cytoskeletal proteins, insufficient resolution of captured images, and outdated algorithms in artificial intelligence models have all become key constraints on the practical application of this technology.

**Table 1:** The quintessential methodologies for biomechanical investigations

Technology category		Principle	Application	Datatype	Invented year
Micropipette aspiration [140]		Assessing the biomechanical properties of cells and tissues through the application of controlled suction to a localized area of the sample utilizing a micropipette	It is commonly employed in the field of cell biomechanics and is particularly useful for studying cellular deformability, adhesion, and viscoelastic properties	Cell deformability, adhesion, and viscoelasticity	1954
Microscopy	Atomic-force microscopy [138, 139]	Measuring the deflection of the cantilever, the forces between the tip and the sample can be quantified	Observe and manipulate the surface of materials at the atomic and molecular scale, provides detailed topographical information	Surface roughness, mechanical properties, and electrical conductivity. stiffness, adhesion, and elasticity of the sample	1986
	Traction-force microscopy [141]	Fluorescent microbeads or markers are embedded within a soft substrate, such as a hydrogel, to which cells can adhere. Deformations in the gel caused by the forces exerted by the cells can then be visualized and quantified using microscopy techniques	Measure the forces exerted by cells on their surrounding substrate or extracellular matrix. It provides insights into the mechanical interactions between cells and their environment	Cell migration, cell mechanics, and cell-substrate interactions	1981
Tweezer	Optical tweezers [142]	Optical tweezers utilize radiation pressure, generated by the transfer of momentum between photons and matter, to measure piconewton-scale forces by tracking the displacement of trapped particles	Use the force of a highly focused laser beam to trap and manipulate microscopic particles	Study forces involved in biological processes, such as molecular motors, protein folding, and cell mechanics	1986
	Magnetic tweezers [143]	Manipulate and apply forces to microscopic objects or particles using magnetic fields0	Allow for the precise control and manipulation of particles at the micrometer or nanometer scale	Study forces involved in biological processes, such as molecular motors, protein folding, and cell mechanics	1949
Probe	Molecular tension probes [144]	Molecular tension probes are typically designed with a force-sensitive domain that undergoes conformational changes in response to mechanical forces. These changes lead to modifications in the fluorescence properties of the probe, enabling the detection and quantification of forces	Förster Resonance Energy Transfer (FRET) probes, fluorescent protein tension probes and DNA-based probes biophysical research to measure and visualize mechanical forces at the molecular level within living cells or tissues	These probes enable researchers to study the forces exerted by cells during various biological processes, such as cell migration, cell–cell interactions, and tissue morphogenesis	2011
	Biomembrane force probes [145]	Biomembrane force probes are designed with a molecularly engineered cantilever, commonly coated with a ligand or functionalized tip that interacts with specific molecules or receptors on the cell membrane	Measure the forces involved in mechanical interactions between cells and their surrounding biomembranes	The mechanical properties and forces associated with cell adhesion, cell membrane mechanics, and cell–matrix interactions	1995
Microfluidic chips [146]		In microfluidic chips, shear stress is generated as fluids flow through microchannels or across interfaces, exerting forces on the surrounding structures or cells	Controlling the magnitude and duration of shear stress can have significant effects on the behavior and responses of cells, particles, and molecules within the system	The magnitude and distribution of shear stress within the chip	1992
4-dimensional flow magnetic resonance imaging [147]		It is based on phase-contrast magnetic resonance imaging. By modifying the intensity and direction of the gradient magnetic field, the velocity of the fluid can be encoded, enabling the measurement of fluid flow velocities in various directions, and providing time-resolved three-dimensional volume information	This allows for the creation of color-coded representations that indicate shear stress within the imaged blood vessels	The magnitude and spatial distribution of shear stress within the imaged blood vessels	1980s
Parallel plate flow chamber [148]		It consists of two parallel plates with a narrow gap between them, through which a fluid is passed. The flow of the fluid creates shear stress on the surfaces of the plates	The magnitude and distribution of shear stress	Observation of cell adhesion, growth, etc. under well-defined shear stress	1985

We have previously discussed the significant role of the ECM in biomechanical research within the TME. To simplify models, researchers have developed hydrogels to simulate the ECM accurately [155]. Hydrogels consist of a three-dimensional network of hydrophilic polymers characterized by high swelling capacity, flexibility, and biocompatibility. They can be designed to possess varying mechanical and biochemical properties by modifying their functional groups or incorporating natural polymers. Among these, polydimethylsiloxane and polyacrylamide hydrogels are two widely used matrices for simulating the ECM [155, 156]. Researchers often modify hydrogels according to their specific research objectives, such as employing micropatterning to create precise and intricate patterns at the micro- or nanometer level to study the stimulation of mechanical forces from the ECM on tumor cells [157]. In addition, the use of force-deformable spherical hydrogel particles allows for the calculation of traction forces on the basis of changes in the shape of the particles [158]. Vorselen et al. successfully utilized this technique to measure dynamic traction forces during the phagocytosis process and when IS with cytotoxic T cells are formed [158].

**Fig. 5:**
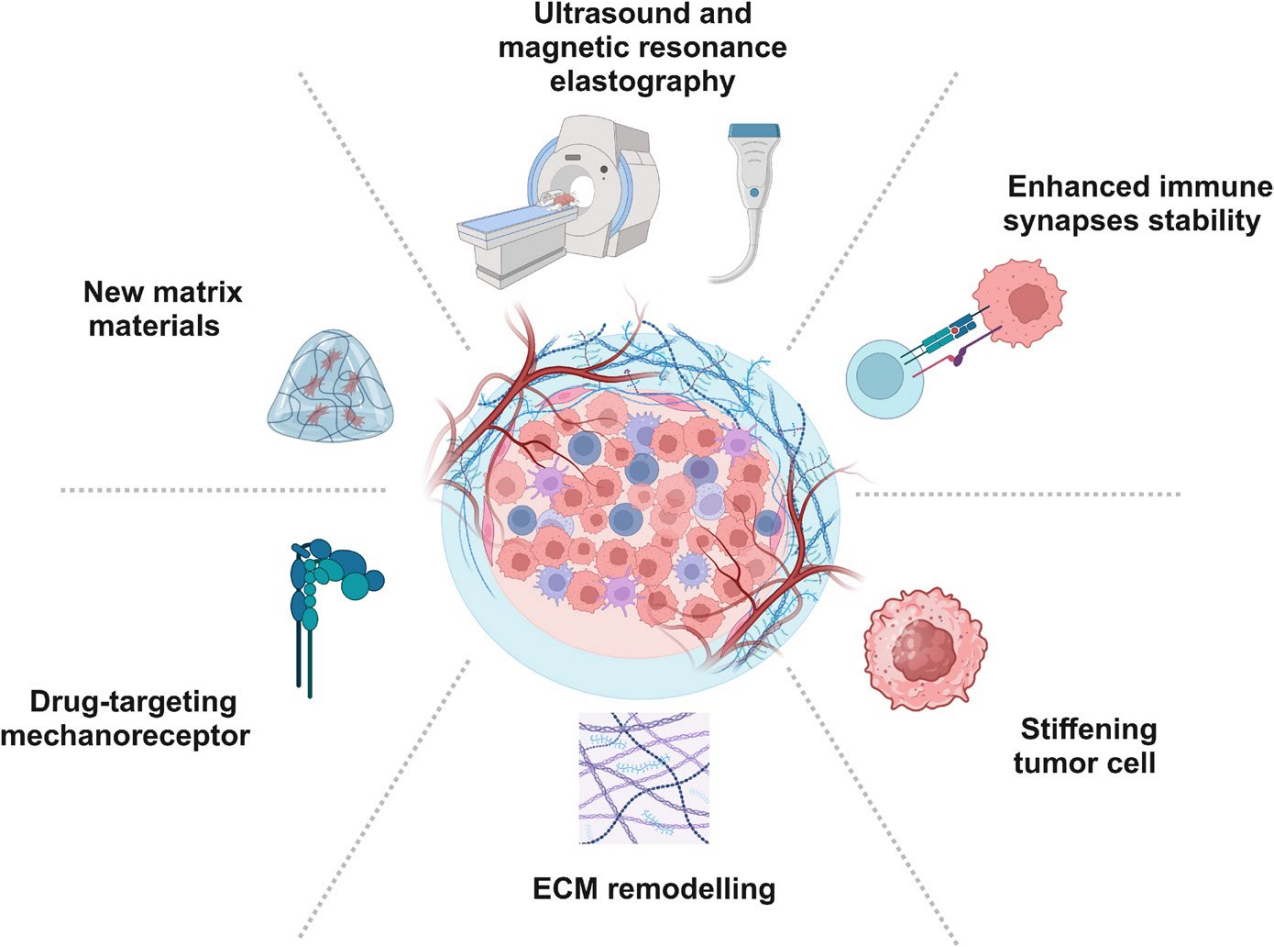
Diagnosis and treatment based on the mechanical characteristics of tumor and immune cells. Magnetic resonance and ultrasound elastography can help detect changes in tumor stiffness. Enhancing the stability of the immune synapse and tumor cell stiffening can improve the efficacy of tumor immunotherapy. Remodeling the ECM and the use of novel matrix materials can facilitate immune cell infiltration and drug release. Combination therapy targeting mechanosensors can improve tumor prognosis. *ECM* extracellular matrix

In addition to cellular measurements, techniques for assessing tissue stiffness have also been developed. Pulsed stimulated Brillouin microscopy enables high-sensitivity elastic imaging of living and delicate biological samples [159]. Ultrasound elastography and magnetic resonance elastography have been employed as non-invasive quantitative imaging techniques for the clinical assessment of soft tissue elasticity and structure (Fig. 5). These methods can be used to monitor diseases with significant stiffness changes, such as liver fibrosis and prostate tumors [160–162]. Direct measurements through elastic imaging have revealed that matrix stiffness is associated with poorer pathological complete response (CR) and reduced disease-free survival [163]. However, the biomechanical measurement techniques currently applied in clinical practice remain in their infancy. This is partly due to the absence of established mechanical indicators that are linked to tumor diagnosis, grading, and prognosis. Additionally, the complexity of human tumors and the challenges associated with developing relevant technologies necessitate further exploration in the field of tumor biomechanics.

## Clinical implications from biomechanics in the TME

### Pre-clinical evidence

As previously mentioned, the stiffness of tumor cells is crucial for their stemness, metastasis, and corresponding tumor immune responses. Therefore, increasing the stiffness of cancer cells may represent an effective therapeutic strategy (Fig. 5). The stiffness of cells is primarily maintained by the cytoskeleton, which includes microtubules, microfilaments, and intermediate filaments. It has been shown that overexpressing MRTFs can effectively increase the density of microfilaments in tumor cells, thereby increasing cancer cell stiffness [98]; however, specific agonists for this target have yet to be identified. Currently, several drugs, including jasplakin, 4-hydroxyacetophenone, nocodazole, and salinomycin, have been shown to increase actin polymerization and the stiffness of cancer cells. Multiple studies have shown that cancer cell migration and invasion are significantly inhibited after treatment with these drugs [99, 164–166]. However, the existing evidence has been derived primarily from in vitro cell experiments, and these drugs can also significantly impair the functions of immune cells, stromal cells, and other normal cells, which limits their clinical application. Chemotherapy is one of the most common treatment methods for tumors, and microtubule-targeting agents (MTAs), such as paclitaxel, are widely used as chemotherapeutic drugs [167]. The anticancer mechanisms of MTAs have focused mainly on their ability to disrupt cancer cell mitosis, thereby inhibiting tumor cell proliferation. Recently, it was discovered that they can also significantly increase cancer cell stiffness and enhance Piezo2 signaling responses [167, 168]. Kaffas et al. utilized low-frequency shear modulus measurements and reported that the viscous modulus of cancer cells significantly increased after paclitaxel treatment [169]. This leads to impairments in cancer cell migration and polarization, as well as their ability to adapt to the ECM [170]. However, further in-depth research is needed to better leverage the effects of MTAs in enhancing anticancer efficacy while mitigating the side effects of chemotherapy.

Reducing the cholesterol content in tumor cell membranes represents another efficacious strategy for cancer cell suppression [100, 171, 172]. Methyl-β-cyclodextrin, a targeted cholesterol-depleting agent, has been extensively evaluated across various neoplasms and has been shown to enhance antitumor immunity by increasing cancer cell stiffness [100, 173]. Furthermore, graphdiyne oxide-mediated photodynamic therapy (PDT) can also increase tumor cell stiffness by eliminating ROS and reducing membrane cholesterol accumulation, thereby potentiating T-cell immunological responses [174].

Given that increased ECM stiffness within the TME significantly contributes to tumor metastasis and immunosuppression, reducing ECM rigidity has emerged as a promising therapeutic approach. LOX, a crucial enzyme facilitating collagen formation and ECM stiffening, has been extensively validated as a significant therapeutic target for enhancing both the efficacy of chemotherapy and PD-1 blockade [110, 175]. Liu et al. demonstrated that TGF-β inhibition reduces type I collagen levels and normalizes the ECM, thereby enhancing TME penetration of small-molecule chemotherapeutics and nanomedicines [176]. Additionally, the use of a collagozome, a 100 nm liposomal formulation encapsulating collagenase that directly targets and degrades collagen, could reduce pancreatic tumor desmoplasia and enhance paclitaxel penetration, thus improving chemotherapeutic outcomes [177].

Mechanoreceptors, which serve as crucial intermediaries between ECM stimuli and tumor/immune cells in the TME, present novel therapeutic opportunities through their ability to suppress tumor proliferation and metastasis and enhance immune infiltration. DDR1/2 inhibition augments CD4^+^ and CD8^+^ T-cell infiltration through the suppression of IL-18 synthesis while reducing ECM stiffness, thereby prolonging the survival of tumor-bearing mice [178, 179]. Both HCC and glioblastoma models have validated the efficacy of Piezo1 blockade in promoting antitumor immunity and attenuating tumor progression [25, 180]. TRPV4, a mechanosensitive receptor whose oncological implications remain incompletely understood, requires further investigation to fully comprehend its functional significance. Interested readers can read two exemplary reviews that comprehensively delineate the therapeutic applications and clinical trials of TRPV4 antagonists and agonists in oncological interventions [63, 181]. Integrins are considered important targets in tumor therapy. In another review, Liu et al. summarized existing animal experiments and clinical studies that target integrins [182]. In animal models, integrin activation induces downstream signaling pathways, such as Src, Syk, ALK, PI3K, ERK, and FAK, which contribute to tumor resistance to chemotherapy, radiotherapy, and molecularly targeted therapies. The use of integrin inhibitors can overcome or delay resistance [182]. Furthermore, targeting integrins has demonstrated significant potential in combination immunotherapies, such as immune checkpoint blockade, Chimeric antigen receptor T-cell (CAR-T) immunotherapy, and oncolytic viruses [182, 183].

YAP/TAZ is one of the most extensively pursued biomechanical targets in clinical drug development. Over 50 compounds have been reported to inhibit YAP/TAZ activity [184, 185]; however, current drugs targeting YAP/TAZ fail to address a critical challenge: how to inhibit biomechanical signals regulated by YAP/TAZ without adversely affecting other cellular activities. Consequently, issues such as nephrotoxicity, lack of specificity, and difficulty in identifying patients responsive to YAP/TAZ inhibitors have limited their clinical application [185].

### Clinical trials

Integrins are currently the most established biomechanical targets in cancer therapy (Table 2).Cilengitide is a potent and selective inhibitor of the α5β3 and α5β5 integrin receptors. A phase I clinical study examining the combination of cilengitide with chemotherapy for the treatment of non-small cell lung cancer demonstrated an acceptable toxicity profile and encouraging clinical outcomes [186]. Similar results were observed in a phase I clinical trial investigating cilengitide in combination with paclitaxel for solid tumors [187]. However, a phase III clinical trial evaluating cilengitide, temozolomide, and radiotherapy in patients with MGMT promoter-methylated glioblastoma did not show any benefit compared with the control group [188]. This may be attributed to the dual roles of integrins in tumor progression and the compensatory effects among different integrins. Therefore, before selecting integrin-targeted therapy for cancer, comprehensive analyses of next-generation sequencing, transcriptomic sequencing, and proteomic data are necessary to identify patients suitable for integrin-targeted therapies [182].

**Table 2:** Principal clinical trials addressing therapeutics targeting Integrins (clinicaltrials.gov)

Target	Type	Drug	NCT number	Study status	Disease	Phases	Result	Start year
Integrin	α5β3 and α5β5 integrin inhibitors	Cilengitide	NCT01118676 [186]	Completed	NSCLC	I	Median PFS: 14.4 months (95% *CI*, 8.4 to NR). Median OS: 29.4 months (95% *CI*, 11.73 to NR). No adverse events were observed	2010
			NCT00121238 [199]	Completed	PCa	II	Cilengitide was well tolerated but did not induce PSA responses in nmCRPC	2005
			NCT00112866	Terminated	GBM	II	46.1% (12/26) patients achieved 6-month PFS. The overall PFS: 8 weeks (95% *CI*, 4–16). All patients experienced adverse events, but no serious adverse reactions occurred	2005
			NCT01276496 [187]	Completed	BRCA	I	8.3% patients PR, 41.7% patients SD, 50% patients PD after the first 2 cycles of therapy	2010
			NCT00689221 [188]	Completed	GBM	III	Median OS: 26.3 months (95% *CI*, 23.8–28.8) in the cilengitide group; 26.3 months (95% *CI*, 23.9–34.7) in the control group (HR 1.02, 95% *CI*, 0.81–1.29, *P* = 0.86). No overall additional toxic effects with cilengitide treatment	2008
			NCT00842712 [200]	Completed	NSCLC	II	PFS: 6.2 vs. 5.0 (months), HR: 0.72, *P* = 0.085. Median OS: 13.6 vs 9.7 (months), HR: 0.81, *P* = 0.265. Cilengitide was well-tolerated	2009
	α5β1 Integrin inhibitors	ATN-161	NCT00352313	Completed	Recurrent glioma	I/II	No results posted	2006
			NCT00131651	Terminated	RCC	II	No results posted	2005
	α*5*β3 integrin inhibitors	MEDI-522	NCT00684996	Terminated	RCC	I/II	In patients treated with bevacizumab combined with MEDI-522, adverse events were universally observed, with 66.7% (2/3) experiencing severe adverse reactions	2008
	α5β1 Integrin inhibitors	Volociximab	NCT00666692	Completed	NSCLC	I	No results posted	2008
			NCT00369395	Terminated	Metastatic melanoma	II	No results posted	2006
			NCT00401570 [201]	Completed	mPACA	II	The absence of severe toxicities and preliminary activity at the highest dose level(Volociximab 15 mg/kg i.v. per week) warrants further disease-directed studies	2005
	α1, α2, α3, and α5 integrin inhibitors	E7820	NCT05024994 [202]	Completed	Leukemia	II	No patient achieved a response. 1 patient achieved temporary bone marrow CR but no improvement in hematology	2021
			NCT01133990	Terminated	CRC	I/II	In patients undergoing combined treatment with E7820 at 40 mg/day plus FOLFIRI, treatment-emergent adverse events were universally reported	2010
			NCT00309179	Completed	CRC	II	No results posted	2007
			NCT01773421 [203]	Completed	Advanced Solid Tumors	I	37.8% (14/37) patients experienced SD	2011
	α5 Integrin inhibitors	Abituzumab	NCT00848510 [204]	Completed	CRC and OC patients with liver metastases	I	It was tolerable despite hypersensitivity reactions	2009
			NCT01008475 [205]	Completed	mCRC	I/II	It was well tolerated, with a trend towards improvement in OS	2009
	α5 Integrin inhibitors	CEND-1	NCT03517176 [206]	Completed	mPDAC	I	It had an acceptable safety profile, with objective efficacy achieved in 17 patients (59%)	2018
			NCT05042128	Active, not recruiting	mPDAC	II	No results posted	2022
	α5β3 integrin inhibitors	ProAgio	NCT05085548	Recruiting	PACA	I	No results posted	2021
	α4β1 integrin inhibitors	7HP349	NCT04508179 [207]	Completed	Solid tumors	I	It was well tolerated	2020

*CI* confidence interval, *CR* complete response, *PFS* progression-free survival, *OS* overall survival, *PR* partial response, *SD* stable disease, *PD* progressive disease, *NR* not reached, *HR* hazard ratio, *PSA* prostate specific antigen, *NSCLC* non-small cell lung cancer, *BRCA* breast cancer, *CRC* colorectal cancer, *GBM* glioblastoma, *PACA* pancreatic cancer, *PDAC* pancreatic ductal adenocarcinoma, *PCa* prostate cancer, *RCC* renal cell cancer, *mPACA* metastatic pancreatic cancer, *mPDAC* Metastatic pancreatic adenocarcinoma, *nmCRPC* non-metastatic castration-resistant prostate cancer, *OC* ovarian cancer, *FOLFIRI* combined treatment with irinotecan, leucovorin, and 5-fluorouracil

Owing to a relatively superficial understanding of the biomechanics within the TME, the development of related therapeutic targets remains insufficient. Currently, clinical trials based on biomechanical targets except integrins predominantly consist of Phase I and Phase II studies (Table 3). Two clinical trials targeting LOX2 have been reported, including the combination of sintilimab with gemcitabine for the treatment of metastatic pancreatic cancer and sintilimab combined with 5-fluorouracil, leucovorin, and irinotecan for the treatment of metastatic KRAS-mutant colorectal cancer; neither has improved the clinical outcomes of patients [189, 190]. Treatment regimens targeting CAFs have demonstrated promising RR in phase I clinical trials for melanoma and squamous cell carcinoma, as well as in a phase Ib trial for advanced renal cell carcinoma [191, 192]. However, no survival benefits were observed in phase II trials for advanced colorectal cancer or phase I trials for pancreatic cancer [193, 194]. Other comprehensive reviews have summarized strategies targeting CAFs and current research efforts in detail [195, 196]. In summary, targeting CAFs has the potential to achieve some success in solid tumors of specific cancer types, but further evidence from clinical trials is still needed. Currently, clinical trials targeting YAP/TAZ have focused primarily on their toxic side effects and safety and have demonstrated minimal toxicity in various solid tumor studies. However, compared with control therapy, PDT involving the targeted drug verteporfin did not provide survival benefits [197, 198]. This may be attributed to compensatory mechanisms from other biomechanical signaling pathways.

**Table 3:** Principal clinical trials addressing therapeutics targeting mechanical aspects of TME (except Integrins) (clinicaltrials.gov)

Target	Type	Drug	NCT Number	Study Status	Disease	Phases	Result	Start Year
LOX	Pan-LOX Inhibitor	PXS-5505	NCT05109052	Recruiting	HCC	I/II	No results posted	2022
	LOXL2 monoclonal antibody	Simtuzumab	NCT01472198 [190]	Completed	PACA	II	Simtuzumab was well tolerated, but the addition of simtuzumab to gemcitabine did not improve clinical outcomes in patients with mPACA	2011
			NCT01479465 [189]	Terminated	CRC	II	The addition of simtuzumab to FOLFIRI did not improve clinical outcomes in patients with metastatic KRAS mutant CRC	2011
MMP	MMP-2 and MMP-9 inhibitors	S3304	NCT00078390	Completed	NSCLC	I/II	No results posted	2003
			NCT00033215 [208]	Completed	Solid tumors	I	It is safe and well tolerated, with plasma concentrations higher than those required to inhibit MMP-2 and MMP-9	2001
YAP	YAP inhibitors	Verteporfin	NCT02872064 [209]	Completed	BRCA	I/IIa	It is tolerated and the effect is proportional to the dose	2013
			NCT02939274 [210]	Recruiting	mBRCA	II	No results posted	2016
			NCT02464761 [211]	Completed	Cancer with spinal metastases	I	Vertebral photodynamic therapy as an adjunct to vertebral cement augmentation is safe and helps relieve pain	2011
			NCT03033225 [212]	Completed	PACA	II	62.5% (5/8) patients had response to treatment, with no adverse reactions reported among the cohort	2016
		ION537	NCT04659096	Completed	Solid tumors	I	No results posted	2021
YAP; TEAD	YAP inhibitors; TEAD inhibitors	IAG933	NCT04857372	Recruiting	Advanced mesothelioma or other solid tumors with inactivating NF2/LATS1/LATS2 or YAP/TAZ fusions	I	No results posted	2021
TEAD	TEAD inhibitors	VT3989	NCT04665206	Recruiting	Advanced malignant mesothelioma or solid tumor with with inactivating NF2 mutations or YAP/TAZ rearrangements	I/II	No results posted	2021
TEAD	TEAD inhibitors	IK-930	NCT05228015	Active, not recruiting	Advanced solid tumors	I	No results posted	2022
CAF	FAP inhibitors	Sibrotuzumab	NCT02198274 [194]	Completed	CRC	II	It is tolerated but failure due to limited response to treatment	2000
		RO6874281	NCT03063762 [192]	Completed	RCC	I	It has acceptable safety and better clinical activity	2017
			NCT02627274 [191]	Completed	Solid tumors	Ia/Ib	It is safety. Long-term response was observed in 3 patients (18.75%). Tumor shrinkage was observed in 4 melanoma patients	2015
			NCT03875079	Completed	Advanced or metastatic melanoma	Ib	No results posted	2019
	VDR agonist	Paricalcitol	NCT03883919 [193]	Completed	PDAC	I	It was well tolerated. 2 patients (12%) had an objective response DCR: 65%, The median PFS for all patients: 3.57 months (95% *CI* 1.28–8.12), The median OS: 6.14 months (95% *CI* 2.43–11.77)	2019
			NCT00634582	Terminated	PCa with bone metastases	II	All patients (2/2) experienced adverse events, with one patient suffering serious adverse events	2009
			NCT04054362	Recruiting	mPDAC	II	Of the 16 patients treated with paricalcitol and chemotherapy, ORR: 0% (CR + PR). At 9 weeks, ORR: 21.4% (CR + PR + SD). The addition of paricalcitol resulted in a PFS of 1.6 months and an OS of 4.8 months [213]	2018
			NCT03415854	Completed	PDAC	II		2018
			NCT03520790	Active, not recruiting	PDAC	I/II	No results posted	2018
			NCT02754726 [214]	Active, not recruiting	mPDAC	II	Among the 24 patients, 19 achieved PR, 2 had SD, and 2 had PD, yielding ORR of 83%. The median PFS: 8.17 months; the median OS: 15.3 months	2016
			NCT03331562	Completed	PDAC	II	No results posted	2017
			NCT04617067	Completed	PDAC	II	No results posted	2020

*CI* confidence lnterval, *CR* complete response, *PFS* progression-free survival, *OS* overall survival, *PR* partial response, *SD* stable disease, *PD* progressive disease, *ORR* overall response rate, *DCR* disease control rate, *BRCA* breast cancer, *mBRCA* metastatic breast cancer, *HCC* hepatocellular carcinoma, *NSCLC* non-small cell lung cancer, *CRC* colorectal cancer, *PCa* prostate cancer, *PACA* pancreatic cancer, *mPACA* metastatic pancreatic cancer, *PDAC* pancreatic ductal adenocarcinoma, *mPDAC* metastatic pancreatic adenocarcinoma, *RCC* renal cell cancer, *FOLFIRI* Combined treatment with irinotecan, leucovorin, and 5-fluorouracil,

## Concluding remarks

With the continuous advancements in interdisciplinary technologies such as biophysics and biochemistry, our understanding of the TME is being refined. Over the past decade, we have gradually recognized that the biomechanical characteristics of the TME represent a new hallmark of cancer. The varying stiffness of tumor cells reflects their different states and fates: low stiffness is associated with enhanced metastatic potential and stemness, whereas high stiffness indicates increased colonization ability and proliferative potential of tumor cells. Tumor cells within the TME do not exist in isolation; they constantly engage in "physical signaling" communication with the ECM and other stromal and immune cells. These signals trigger the activation of mechanical receptors, which subsequently activate downstream signaling pathways and actively remodel their own and ECM stiffness, leading to diverse immune responses. Soft tumor cells are rarely involved in these biomechanical pathways, thus evading immune destruction and facilitating invasion and metastasis. Conversely, a stiff ECM can accelerate the exhaustion of T and NK cells, limit their infiltration, and promote invasion and metastasis by mediating EMT in tumor cells. Although targeting these mechanoreceptors and associated signaling pathways has shown promising anticancer effects in preclinical models, more robust evidence from clinical trials is needed to substantiate their efficacy. In summary, research on the biomechanics of the TME is currently limited by insufficient technological methods and is still in its infancy. Both fundamental research and clinical trials should progress concurrently to break new ground in cancer research and inspire novel therapeutic strategies.

## Data Availability

No datasets were generated or analysed during the current study.

## Abbreviations

AFM: Atomic force microscopy
AGEs: Advanced glycation end-products
APCs: Antigen-presenting cells
CAR-T: Chimeric antigen receptor T-cell
CSCs: Cancer stem cells
CR: Complete response
cSMAC: Central central supramolecular activation cluster
CTCs: Circulating tumor cells
DDRs: Discoidin domain receptors
DsDNA: Double-stranded DNA
ECM: Extracellular matrix
EMT: Epithelial-mesenchymal transition
FA: Focal adhesion
F-actin: Filamentous actin
FSS: Fluid shear stress
HA: Hyaluronic acid
HCC: Hepatocellular carcinoma
IS: Immune synapses
LOX: Lactate oxidase
MMP: Matrix metalloproteinases
MTAs: Microtubule-targeting agents
MTOC: Microtubule organizing center
PDT: Photodynamic therapy
PCa: Prostate cancer
ROS: Reactive oxygen species
RR: Response rate
RTKs: Receptor tyrosine kinases
TCR: T cell receptor
TGF-β: Transforming growth factor-β
TME: Tumor microenvironments
